# Clinically assisted nutrition and hydration at the end of life

**DOI:** 10.1016/j.clinme.2025.100323

**Published:** 2025-05-08

**Authors:** Andrew Davies

**Affiliations:** a Trinity College Dublin, School of Medicine, Dublin, Ireland; b University College Dublin, School of Medicine, Health Sciences Centre, Belfield, Dublin, Ireland; c Our Lady’s Hospice & Care Services, Academic Department of Palliative Medicine, Harold’s Cross, Dublin, Ireland

**Keywords:** Palliative medicine, Palliative care, Clinically assisted hydration, Clinically assisted nutrition

## Abstract

The initiation, continuance or discontinuance of clinically assisted hydration and nutrition are some of the most challenging decisions in patients near the end of life. This article reviews the limited evidence to support or otherwise these medical treatments, and provides an overview of relevant clinical practice guidance. Essentially, decisions need to be individualised, and importantly regularly reviewed to ensure that the objectives of treatment are being achieved.

**Key Points**

 

• Decisions about initiating, continuing and discontinuing clinically assisted hydration (CAH) / nutrition (CAN) are challenging in patients near the end of life.
• The evidence base to support decision making around CAH / CAN is lacking (in amount / quality).
• Decision making around CAH / CAN needs to be individualised (and involves careful assessment and reassessment).
• Contemporary clinical guidance is available to support decision making.
• Separate decision making is required for the two medical treatments.

Alt-text: Unlabelled box

## Introduction

The initiation, continuance or discontinuance of clinically assisted hydration and nutrition are some of the most challenging decisions in patients near the end of life. The challenges relate to paucity of evidence, and the widespread perception of the benefits of giving these medical treatments, and the correlated negative effects of withholding/withdrawing them. This article will review the current evidence for clinically assisted hydration and nutrition, and provide an overview of current clinical guidance on the utilisation of these medical treatments.

## Clinically assisted hydration (CAH)

CAH involves the provision of fluids via the intravenous route, subcutaneous route (‘hypodermoclysis’), or via a tube inserted into the stomach, duodenum, jejunum or rectum (‘proctoclysis’).^1^ CAH is used to treat dehydration and/or maintain hydration in patients with inadequate oral intake, or excessive fluid losses (eg patients with malignant bowel obstruction).

The Cochrane systematic review of medically assisted hydration (MAH; synonymous with CAH) in patients receiving palliative care concluded that ‘there remains insufficient evidence to determine whether MAH improves QoL *(quality of life)* or prolongs survival, compared with placebo or standard care’.^2^ The review identified four randomised controlled trials (RCTs), involving a total of only 422 patients. Importantly, three of these RCTs involved dehydrated patients and suboptimal fluid therapy, ie insufficient quantities to reverse dehydration.^3^

The survival ‘rule of threes’ states that a person cannot live for more than 3 days without water, although there appears to be no evidence to corroborate this statement in the medical literature. In patients with a prognosis of weeks to months (or more), the decision to commence CAH is relatively straightforward: withholding CAH in these circumstances is likely to lead to death from dehydration rather than the underlying condition.^4^ However, in patients with a prognosis of days, the decision to withhold CAH is much more challenging (and needs to be individualised).

The Palliative Care Study Group of the Multinational Association of Supportive Care in Cancer (MASCC) has produced clinical guidance on CAH in patients with advanced cancer,^5^ although the principles can be applied to other groups of patients with life-limiting conditions. The guidance features 12 expert opinion statements (Table 1), together with a number of clinical considerations (Table 2), and a treatment decision algorithm based upon estimated prognosis. The MASCC guidance also includes a number of legal/ethical principles, which equally apply to decisions relating to the provision of clinically assisted nutrition (Table 3). It should be noted that the evidence to support these recommendations was somewhat limited (and generally of relatively low quality).

**Table 1:** MASCC recommendations regarding clinically-assisted hydration [reproduced from reference^5^].

Assessment / decision making
— All patients with advanced cancer should be regularly assessed regarding hydration/dehydration.
— Decisions relating to clinically assisted hydration (CAH) should be made by an appropriately constituted multidisciplinary healthcare team together with the patient and their family.
— All patients receiving CAH should be regularly reassessed.
Management (generic)
— CAH should be considered in patients at risk of dying from dehydration before dying from their cancer.
— Patients receiving CAH should have a hydration care plan which defines the agreed objectives of treatment, and the agreed conditions for withdrawal of treatment.
— Protocols/processes should exist to deal with conflicts over the initiation (or withdrawal) of CAH.
Management (specific)
— Patients should be practically supported to maintain oral intake.
— Reversible causes of decreased fluid intake, or increased fluid loss, should be treated.
— Patients should be given fluids via the most appropriate route (for that patient).
— CAH should be available in all settings, including the home setting.
— Patients who are dehydrated should be given sufficient fluids to reverse the dehydration.
— Patients who are not dehydrated should be given sufficient fluids to maintain hydration / prevent dehydration.

**Table 2:** Clinical considerations relating to decisions about clinically-assisted hydration [reproduced from reference^5^].

Clinical considerations
• Patient’s views.
• (Family’s views).
• Estimated prognosis.
• Current hydration status.
• Oral intake.
• Fluid losses.
• ‘Hydration impact symptoms/problems’.
• Comorbid conditions (eg cardiac disease, renal impairment).
• Suitability of routes of administration.
• Availability of indwelling catheters / enteral feeding tubes.
• Current/future place of care.

**Table 3:** Legal / ethical considerations relating to decisions about clinically-assisted hydration (and clinically-assisted nutrition) [reproduced from reference^5^].

Legal / ethical considerations
• The physician/multidisciplinary team has the ultimate responsibility for making the decision on clinically assisted hydration (CAH).
• CAH should be considered if the potential benefits outweigh the potential burdens (and vice versa).
• CAH should be considered if it is unclear whether the potential benefits outweigh the potential burdens (ie give a trial of CAH).
• The patient does not have the right to demand CAH.
• The patient does have the right to refuse CAH (if the patient has capacity/competence).
• A valid advance directive to refuse treatment must be followed (if the patient does not have capacity/competence).
• The family do not have the right to demand CAH.

As discussed, the initiation, continuance or discontinuance of CAH is challenging in patients near the end of life. In some cases, there are clear indications for providing CAH (eg thirst, opioid toxicity), while in other cases there are clear contraindications to providing CAH (eg fluid overload, syndrome of inappropriate antidiuretic hormone secretion).^1^ However, in most cases the decision is highly subjective. Importantly, healthcare professionals need to understand that many common assertions about CAH are either unproven or actually mistaken. For example, a general ‘concern’ relates to the development/worsening of audible upper airway secretions (aka ‘death rattle’). However, data from research studies suggests no link between CAH/hydration status and death rattle.^6^^,^^7^

Of note, the General Medical Council’s ‘Treatment and care towards the end of life: good practice in decision making’ provides guidance on dealing with differences in opinion relating to the provision of CAH (and other medical treatments) in patients at the end of life.^8^

## Clinically assisted nutrition (CAN)

CAN involves the provision of food via the intravenous route (parenteral nutrition), or via a tube inserted into the stomach, duodenum or jejunum (enteral nutrition). CAN is generally indicated in patients with reversible/irreversible inability to ingest sufficient nutrients (but not secondary to anorexia), or in patients with reversible/irreversible inability to absorb sufficient nutrients.^9^ Moreover, CAN is generally not indicated in patients with cancer cachexia (or isolated anorexia/weight loss), or in patients with ‘nutrition impact symptoms’ causing reduced oral intake (eg dry mouth, taste disturbance, oral discomfort, dental/denture problems).^9^

The ‘stable’ Cochrane systematic review of medically assisted nutrition (synonymous with CAN) in patients receiving palliative care concluded that *‘*there are insufficient good-quality studies to make any recommendations for practice with regards to the use of medically assisted nutrition in palliative care patients’.^10^ The review identified no RCTs, but reported four uncontrolled studies (involving a total of only 360 patients).

In contrast to dehydration, there are good data on the effect of starvation on survival (at least in healthy young males. Thus, the Irish Republican Army (IRA) hunger strikers survived for a mean of 61 days (range 46–73 days).^11^ However, survival would be expected to be somewhat reduced in patients with advanced cancer, and CAN should generally be considered in patients with an estimated prognosis of more than a month, unless there are particular indications such as ongoing hunger.^9^

The Palliative Care Study Group of MASCC have also produced guidance on CAN in patients with advanced cancer,^9^ although the principles can again be applied to other groups of patients with life-limiting conditions. The guidance features 12 recommendations (Table 4), together with a number of clinical considerations, a number of legal/ethical principles (Table 3), and a treatment decision algorithm based upon estimated prognosis. Again, it should be noted that the evidence to support these recommendations was somewhat limited (and generally of relatively low quality).

**Table 4:** MASCC recommendations regarding clinically assisted nutrition [reproduced from reference^9^].

Assessment / decision making
— All patients with advanced cancer should have regular nutritional assessments.
— Patients with nutritional problems should be reviewed by a specialist dietitian (with/without other members of the nutrition support team).
— Any decision to initiate clinically assisted nutrition (CAN) should be made by an appropriately constituted multidisciplinary healthcare team together with the patient and their family.
— All patients receiving CAN should be regularly reassessed.
Management (generic)
— CAN should be considered in patients at risk of dying from malnutrition before dying from their cancer.
— Patients receiving CAN should have a nutritional care plan which defines the agreed objectives of treatment, and the agreed conditions for withdrawal of treatment.
— Protocols/processes should be in place to deal with conflicts over the initiation (or withdrawal) of CAN.
— CAN is not indicated for the treatment of cancer cachexia.
Management (specific)
— CAN should be considered in patients with an inability (reversible/irreversible) to ingest sufficient nutrients.
— CAN should be considered in patients with an inability (reversible/irreversible) to absorb sufficient nutrients.
— Enteral tube feeding is generally preferable to parenteral nutrition (if possible).
— CAN should be available in all settings, including the home setting.

## Conclusion

The current evidence base regarding CAH/CAN is highly limited, and further (robust) research studies are required to ensure that palliative care patients receive optimal end-of-life care. In the meantime, decision making should be individualised, with treatments regularly reviewed, and whenever necessary altered.

## Declaration of competing interest

The authors declare the following financial interests/personal relationships which may be considered as potential competing interests: Prof Davies is the chief investigator on the CHELsea II trial (cluster randomised trial of clinically assisted hydration in the last days of life) which is funded by the National Institute for Health and Care Research (grant number – 131687).

Prof Davies is a member of the Editorial Board of *Clinical Medicine*.
